# Tree Species Mixing Regulates Soil Multi-Nutrient Cycling by Altering Microbial Network Complexity and Assembly Processes in *Larix olgensis*

**DOI:** 10.3390/microorganisms14020388

**Published:** 2026-02-06

**Authors:** Yue Liu, Chunjing Jiao, Wanju Feng, Yuchun Yang, Bing Yang, Fang Wang, Jun Wang

**Affiliations:** 1 Jilin Provincial Academy of Forestry Sciences, Changchun 130033, China; yueliu0211@163.com (Y.L.);; 2 The Conservation of Endangered Wildlife Key Laboratory of Sichuan Province, Sichuan Academy of Giant Panda, Chengdu 610081, China

**Keywords:** soil multi-nutrient cycling, *Larix olgensis*, rhizosphere soil, network complexity, assembly process, mixed-species plantations

## Abstract

Establishing mixed conifer–broadleaf forests enhances soil multi-nutrient cycling (SMC), yet the underlying mechanisms, particularly the role of rhizosphere microbial communities, remain poorly understood. This study investigated how bacterial and fungal communities in the rhizosphere soil of *Larix olgensis* drive SMC in both pure and mixed plantations with *Fraxinus mandshurica*, elucidating the microbial pathways for nutrient supply in mixed stands. Our results indicated that SMC in the *L. olgensis* rhizosphere soil was significantly greater in mixed stands (0.43) than in pure stands (−0.51). Tree species mixing significantly enhanced microbial diversity, increased the stochasticity of community assembly, and reduced dispersal limitation. Cross-kingdom (bacteria–fungi) co-occurrence networks in mixed stands showed a 19.7% increase in positive correlations, indicating stronger microbial cooperation. Random forest analysis identified microbial diversity, network complexity, and bacterial assembly processes as the main predictors of SMC. Structural equation modeling indicated that microbial diversity indirectly promoted SMC via increased network complexity, while bacterial assembly processes directly influenced SMC. These findings demonstrate that mixed conifer–broadleaf plantations improve soil microbial functioning and nutrient cycling by modifying microbial diversity, assembly processes, and interaction networks.

## 1. Introduction

Forests cover nearly one-third of Earth’s terrestrial ecosystems and play a crucial role in maintaining soil ecological functions, such as nutrient cycling, soil fertility maintenance, and water conservation [1]. China has the largest plantation area in the world, totaling 80 million hectares and accounting for 36% of its total forest cover. However, inadequate plantation management can trigger ecological risks that severely constrain sustainable development [2]. In particular, large-scale, high-density monocultures of coniferous species, such as pure *L. olgensis* stands, can lead to soil fertility degradation, biodiversity loss, and slow ecosystem nutrient turnover, ultimately hindering the sustainable development of plantations [3]. To improve plantation soil quality, many regions have begun implementing silvicultural models, such as mixed conifer–broadleaf plantations, which improve soil nutrient status and ecosystem stability [4]. Compared with conventional methods that focus on individual nutrients, the soil multi-nutrient cycling (SMC) index concurrently accounts for the concentrations of multiple nutrient elements and their interactions, thereby offering a more systematic appraisal of how soils respond to environmental change and of their capacity to sustain ecosystem biodiversity [5]. Moreover, soil microorganisms play a central role in regulating the SMC index [6]. Their physiological status and metabolic functions are highly sensitive to environmental change, and prolonged disturbances can induce nondirectional shifts in functional composition, thereby altering nutrient transformation efficiency and balance [7]. A more precise explanation of the relationship between microbial community structure and the SMC helps to avoid overinterpreting microbial functional redundancy. This enables the direct assessment of the contribution of microbial diversity to SMC [8]. Consequently, understanding the interaction mechanisms between the structure of the microbial community in plantation soils and SMC is crucial for optimizing stand productivity and managing ecosystem health.

However, the relationships between microbial communities and the SMC index remain unclear [9]. Studies have reported negative, positive, or nonsignificant associations between microbial diversity and SMC [10,11,12,13,14]. This discrepancy is because most studies have only examined simple correlations between microbial diversity or community composition and SMC, overlooking the way in which complex interspecific interactions modulate this relationship. Recent studies have demonstrated that microbiome complexity and community assembly processes are pivotal in driving soil ecosystem biogeochemical cycling, energy flow, and information transfer [15]. Microbial network analysis and community assembly processes can reveal complex interspecific interactions (e.g., competition, cooperation, and antagonism) and mechanisms of community formation (deterministic versus stochastic processes). This finding reveals many important ecological processes and functional linkages that diversity metrics alone fail to capture [16]. Therefore, the relationship between microbial diversity and the soil multi-nutrient cycling (SMC) index is likely dependent on microbial network characteristics and community assembly dynamics. Moreover, functional redundancy is widespread within microbial communities. Despite marked differences in species composition, taxa that carry out key metabolic functions often exhibit replacement and compensatory dynamics. While this functional redundancy underpins the temporal stability of ecosystem processes, it also implies that metrics of species richness alone are insufficient to resolve the functional dynamics of microbial communities or to reveal the intrinsic mechanisms by which they govern shifts in the SMC index [17]. Notably, the relationships between microbial community attributes and the SMC index are strongly influenced by environmental factors, such as stand type and tree identity [18], as well as by soil properties, such as soil moisture and pH [19,20]. For instance, research conducted in Chinese fir plantations has revealed that conifer–broadleaf combinations can substantially increase stand-level SMC [6]. However, broadleaf species often have a competitive advantage because of their superior ability to acquire nutrients, which can intensify nutrient competition within mixed stands [6]. In response, conifers typically increase their root biomass and exudation and allocate more carbon belowground. This reshapes the rhizosphere microbial communities and soil nutrient-cycling processes, thereby offsetting their disadvantage in nutrient uptake [21]. While the adaptive strategies of conifers in mixed stands have been partially documented, the specific mechanisms by which they regulate rhizosphere microbial communities to maintain SMC remain unclear. Therefore, it is necessary to systematically assess and quantify the effects of tree species composition on conifer rhizosphere soil properties and microbial community structure. These findings clarify how these changes affect SMC and deepen our understanding of conifer ecological adaptation under strong competition from broadleaf species.

*Larix olgensis* is the most widespread coniferous plantation species in Northeast China, ranking first in both area and stocking volume [22]. However, its slow litter decomposition can lead to soil acidification and a decrease in soil fertility, creating typical “green deserts” [23]. Compared with pure stands, mixed stands with broadleaf species can significantly mitigate these adverse effects [24,25], contributing to improved soil fertility [26,27]. Nevertheless, systematic research on how the rhizosphere SMC of *L. olgensis* responds to changes in stand composition and the underlying microbial mechanisms is still lacking. Therefore, this study aims to investigate the effects of mixing *L. olgensis* with broadleaf species on the SMC in *L. olgensis* rhizosphere soil and to elucidate the potential microbial mechanisms involved. We propose the following hypotheses: (1) Tree species mixing increases the SMC in *L. olgensis* rhizosphere soil, and (2) this increase stems primarily from changes in soil microbial diversity, reflecting the fundamental role of soil microbial communities in regulating nutrient cycling in forest ecosystems [28]. In this study, we focus on how *L. olgensis* in mixed stands affects rhizosphere SMC to cope with resource-competition pressure, and we aim to elucidate the dynamic regulatory mechanisms through which microbial alpha diversity, community assembly processes, and microbial interaction networks influence SMC.

## 2. Materials and Methods

### 2.1. Study Sites

This study was conducted in the protective forest area of the Yongqing Forest Farm (25°33′ N, 116°18′ E), with a mean elevation of 779 m. Meteorological data from 2001 to 2023 were obtained from a nearby weather station via the China Meteorological Data Network (http://data.cma.cn, accessed on 3 April 2025). Analysis revealed that the region’s mean annual temperature over the past two decades (2001–2023) was 3.4 °C, with an average annual precipitation of 867 mm, characterizing a temperate semihumid monsoon climate with distinct seasons: dry and windy springs, warm, humid summers with concentrated rainfall, rapid temperature decreases and frequent early frosts in autumn, and long, cold, dry winters. The vegetation is predominantly composed of tree species such as *Larix olgensis*, *Pinus koraiensis*, and *Fraxinus mandshurica*. Understorey shrubs and herbs include *Syringa reticulata*, *Lonicera japonica*, *Philadelphus schrenkii*, *Euonymus alatus*, *Sambucus williamsii*, and *Rhamnus davurica*. The primary soil type is dark brown forest soil.

Two-year-old *Larix olgensis* seedlings were planted in the spring of 1990 and 2001 at the Yongqing Experimental Forest Farm to establish two stand types: a pure *L. olgensis* plantation (P) and a mixed plantation of *L. olgensis* and *Fraxinus mandshurica* (M). The planting spacing was 1.5 m × 1.5 m, and the plants were arranged in a single row. The stands underwent five tending operations over three years. The site conditions for both forests were comparable, with average slopes of 2° and 3°, respectively.

### 2.2. Vegetation Survey and Soil Sampling

In September 2021, employing a stratified randomized complete block design within the context of the National Forest Conservation Program, we selected protective forest plots (PF-20 and PF-31) established in different years (1990 and 2001) but with similar soil conditions to form a restoration chrono sequence. The experiment followed a complete factorial design: 2 (plantation ages) × 2 (stand types) × 6 (replicates). For each treatment combination, six plots (20 × 20 m) were randomly established, with adjacent quadrats spaced ≥200 m apart to minimize edge effects, resulting in a total of 24 plots. We measured the tree height and diameter at breast height (*DBH*) of *Larix olgensis* in both pure and mixed plantations (Table S1).

From each plot, six representatives of *Larix olgensis* were selected for rhizosphere soil collection. Briefly, live roots were carefully excavated from the topsoil (0–20 cm) and gently shaken by hand. Soil that adhered tightly to the roots (approximately 4 mm thick) was defined as rhizosphere soil [29]. The samples from the six trees per plot were composited into one representative sample per plot. Upon transport to the laboratory, each composite sample was divided into three parts. One part was stored at −80 °C for molecular analysis (qPCR and high-throughput sequencing), the second part was stored at 4 °C for the analysis of available nutrients, and the final part was air-dried, sieved, and used for pH measurement and the assessment of soil organic carbon, available potassium, total nitrogen and total phosphorus.

### 2.3. Soil Physicochemical Analyses and Soil Multi-Nutrient Cycling

We determined soil pH, soil organic carbon (SOC), total phosphorus (TP), total nitrogen (TN), ammonium nitrogen (NH_4_^+^-N), nitrate nitrogen (NO_3_^−^-N), available phosphorus (AP), available potassium (AK), and soil water content (SWC) using standard methods [30,31,32]. Detailed methods for determining soil properties are provided in the Supplementary Materials.

The SMC index, representing multiple nutrient-supply capacities, was calculated using an averaging approach-a straightforward and interpretable method for quantifying multifunctionality [31,33]. Following Jing et al. (2015) and Jiao et al. (2018), SOC, NO_3_^−^-N, NH_4_^+^-N, TN, TP, AP, and AK values were standardized by Z-score transformation and then averaged to obtain an SMC index for each sample [5,31]. Differences in soil physicochemical properties and SMC between the two plantation types and between the two plantation ages were evaluated using the Wilcoxon rank-sum test in R (version 4.3.1). Bacterial and fungal α-diversity indices (including Shannon, Pielou, and Chao1) and β-diversity based on Bray–Curtis distances were assessed using the “vegan” package (version 2.7-2). Permutational multivariate analysis of variance (PERMANOVA) was performed with the Adonis function in vegan, with significance evaluated through 999 permutations, to determine differences in rhizosphere microbial communities of Larix plantations across different stand types. Following these analyses, a random forest (RF) model was applied to evaluate the contributions of microbial parameters to soil multi-nutrient cycling (SMC), using the “rfPermute” package (version 2.5). In addition, the analytical procedures for microbial assembly processes, bacterial–fungal cross-kingdom network construction, and the RF and SEM pathway models are described in detail in the Supplementary Materials.

### 2.4. Sequence Analysis

Rhizosphere soil DNA was extracted from homogenized soil samples (0.5 g) via a FastDNATM SPIN Kit for Soil (MP Biomedicals, Santa Ana, CA, USA) following the manufacturer’s instructions. The DNA concentration and purity were assessed with a NanoDrop 2000 UV–vis spectrophotometer (Thermo Scientific, Wilmington, DE, USA). The quality and quantity of the extracted DNA were assessed via a 1.0% (*w*/*v*) agarose gel [34]. The V 3-V4 region of the bacterial 16S rRNA gene was amplified via the primers 338F (ACTCCTACGGGAGGCAGCA) and 806R (GGACTACHVGGGTWTCTAAT). The ITS1 region of the fungal gene was amplified using specific primer pairs (i.e., ITS1–1F-F: 5′-CTTGGTCATTTAGAGGAAGTAA-3′; ITS1-1F-R: 5′-GCTGCGTTCTTCATCGATGC-3′. The amplicons were subsequently merged and sequenced on the Illumina MiSeq platform (Illumina, San Diego, CA, USA) in equimolar amounts via paired-end sequencing (2 × 300 bp) following the standard protocols provided by Majorbio Bio-Pharm Technology Co., Ltd. (Shanghai, China).

Quantitative Insights into Microbial Ecology (QIIME), which includes quality control, taxonomic assignment, and the generation of operational taxonomic units (OTUs) for bacteria, was utilized [35,36]. Chimeric, single-OTU, and low-quality sequences were excluded manually. OTUs were differentiated at a similarity threshold of 97% via USEARCH and subsequently clustered with UPARSE [37]. The bacterial 16S rRNA sequences were compared with those in the SILVA database (version 138.1, http://www.arb-silva.de/, accessed on 8 May 2025) to assign a taxonomic classification. The resulting OTUs were subjected to comprehensive statistical analysis of the biological data to determine the response of the soil microbes to afforestation. The soil microbial alpha (α) diversity was represented by the Chao 1, Shannon, and Simpson indices in QIIME 2 software  (version 2024.10) [38].

### 2.5. Statistical Analysis

All the statistical analyses were performed using SPSS 22.0 for Windows. The choice of statistical tests was guided by the data structure and underlying assumptions. Two-way analysis of variance (ANOVA) was employed to assess the statistical significance of the effects of mixing (stand type), tree species, and their interaction on soil chemical properties and other variables. The links between soil physicochemical properties, microbiome variables, single soil properties, and SMC were evaluated via ordinary least squares (OLS). Prior to performing two-way ANOVA and OLS regression, the assumptions of normality and homogeneity of variances were verified using the Shapiro–Wilk test and Levene’s test, respectively. All analyses met or approximately met the fundamental assumptions of the parametric tests. For all OLS models, we inspected residual normality (Shapiro–Wilk test and Q-Q plots), homoscedasticity (residuals vs. fitted values), and multicollinearity among predictors using variance inflation factors (VIF), and predictors with VIF > 5 were not included in the same model. When a significant interaction between the mixing effect and tree species effect was detected, differences between the two forest types were further analyzed using one-way ANOVA followed by Duncan’s multiple range test. Relationships between soil properties and microbial properties were examined using Pearson’s correlation analysis. Statistical significance was accepted at a level of *p* < 0.05.

## 3. Results

### 3.1. Changes in Rhizosphere Soil Properties and SMC

**Figure 1:**
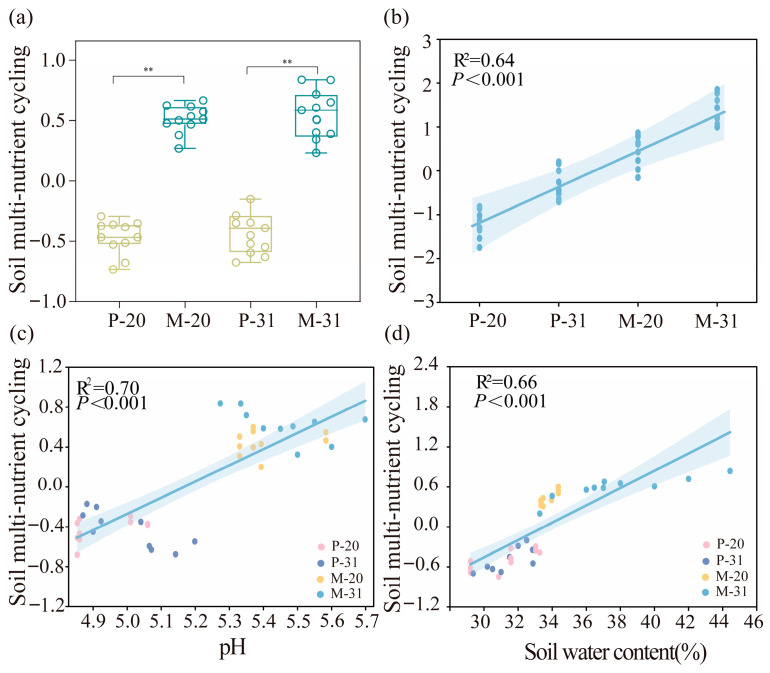
Soil multi-nutrient cycling among different *L. olgensis* plantations (**a**) and the relationships between soil multi-nutrient cycling and forest type (**b**), soil water content (**c**) and pH (**d**). Note: P, the *L. olgensis* in pure plantations; M, the *L. olgensis* in mixed plantations; 20: 20-year-old stands; 31: 31-year-old stands. Within each boxplot, the colors are used to distinguish the treatment conditions: yellow corresponds to the P and cyan corresponds to the M (**a**). ** exhibit significance at the levels of *p* < 0.01 respectively.

Tree species mixing significantly affected the contents of NH_4_^+^, NO_3_^−^, total nitrogen (TN), total phosphorus (TP), available potassium (AK), available phosphorus (AP), soil organic carbon (SOC), and soil pH in the *L. olgensis* rhizosphere soil (Table S2, Figure S1). Stand age also significantly influenced the contents of NH_4_^+^, NO_3_^−^, TN, AK, AP, and SOC. Overall, compared with the pure plantation (P), the mixed plantation (M) experienced significant (*p* < 0.05) or highly significant (*p* < 0.01) increases in the contents of NH_4_^+^, TN, TP, AK, AP, and SOC, while the pH and NO_3_^−^ concentration were significantly lower (*p* < 0.05 or *p* < 0.01). Significant interactive effects of stand age and mixing were observed for SOC, TN, AK, AP, NO_3_^−^, and NH_4_^+^ in the *L. olgensis* rhizosphere soil (*p* < 0.05) (Figure S1). Furthermore, the SMC index was significantly greater in M (0.43) than in P (−0.51) (*p* < 0.01; Figure 1a,b). Further correlation analysis and ordinary least-squares (OLS) regression were employed to quantify the relationships between SWC, pH and individual nutrient cycles (Figure S2), as well as the relationships between the SMC index and SWC and pH (Figure 1c,d). Across all the stands, SMC was significantly and positively correlated with both pH (*R*^2^ = 0.70, *p* < 0.001; Figure 1c) and SWC (*R*^2^ = 0.56, *p* < 0.001; Figure 1d).

### 3.2. Changes in Rhizosphere Soil Microbial Diversity and Composition

**Figure 2:**
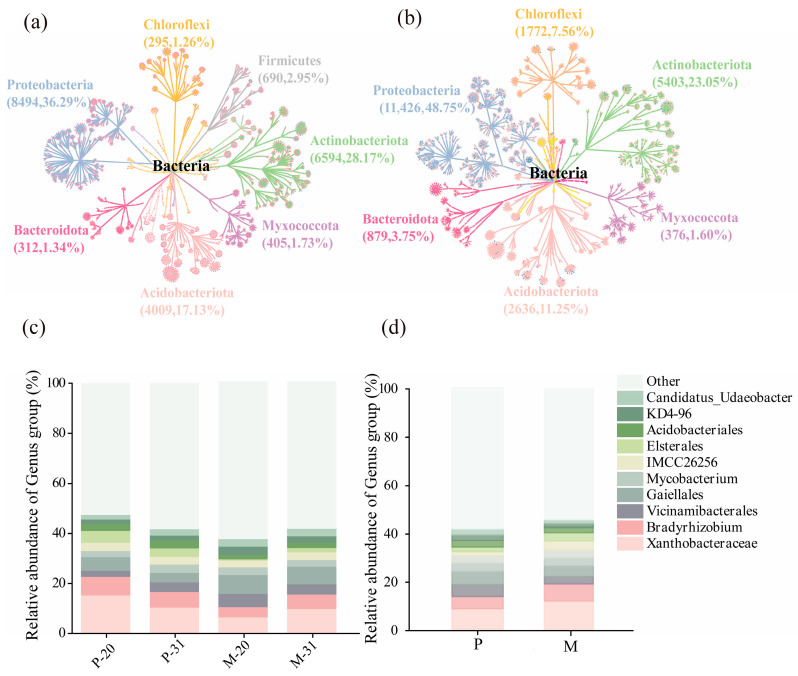
Taxonomic dendrograms and Grouped Stacked Columns of the detected bacterial communities display the OTUs distribution. Each point represents the corresponding classification level. (**a**,**b**) Phylum-level taxonomic composition of bacterial communities in *L. olgensis* conifer rhizosphere soil in pure plantations (**a**) and mixed plantations (**b**). (**c**,**d**) Genus-level taxonomic composition of bacterial community in conifer rhizosphere soil of *L. olgensis* in pure plantations and mixed plantations: The top 11 genera in terms of relative abundance. P, the *L. olgensis* in pure plantations; M, the *L. olgensis* in mixed plantations; 20: 20-year-old stands; 31: 31-year-old stands; Same below.

Following tree species mixing, the relative abundances of r-strategist bacterial taxa, such as Proteobacteria, Actinobacteria, and Bacteroidota, in the *L. olgensis* rhizosphere increased by 27.39%, 7.73%, and 92.3%, respectively. In contrast, the relative abundances of K-strategist bacterial taxa, such as Acidobacteria and Chloroflexi, decreased by 10.30% and 17.54%, respectively (Figure 2a,b). At the genus level, the relative abundances of Bryobacter (P: 7.47%, M: 15.83%), Bradyrhizobium (P: 5.61%, M: 11.37%), and Faecalibacterium (P: 2.61%, M: 5.37%) were significantly greater in the mixed plantation than in the pure plantation (Figure 2c,d).

**Figure 3:**
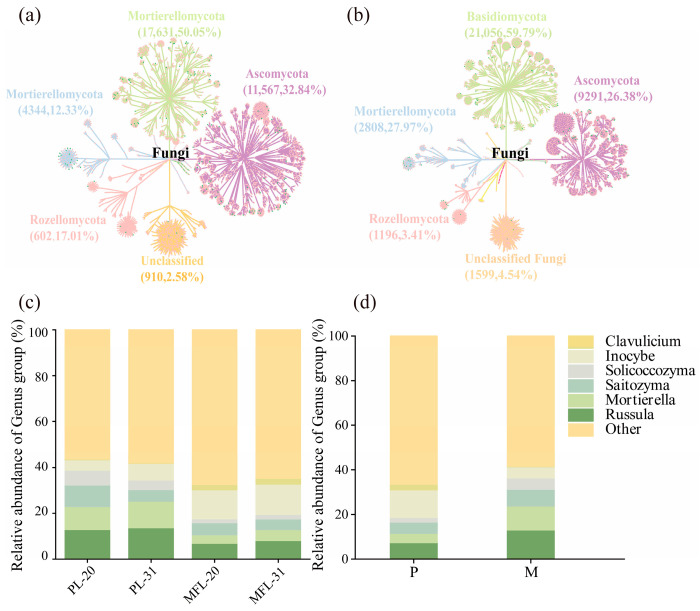
Taxonomic dendrograms and Grouped Stacked Columns of the detected fungal communities display the OTUs distribution. Each point represents the corresponding classification level. Note: (**a**,**b**) Phylum-level taxonomic composition of fungal communities in conifer rhizosphere soil in pure plantations (**a**) and mixed plantations (**b**). (**c**,**d**) Genus-level taxonomic composition of fungal community in conifer rhizosphere soil in pure plantations and mixed plantations: The top 11 genera in terms of relative abundance.

Within the fungal community, the dominant phyla were Ascomycota, Basidiomycota, and Mortierellomycota. The relative abundance of Basidiomycota was lower in the mixed plantation (M: 40.03%) than in the pure plantation (P: 50.45%) (Figure 3a,b). Analysis at the genus level revealed that the relative abundances of Russula (P: 7.37%, M: 13.01%), Mortierella (P: 4.18%, M: 10.83%), and Saitozyma (P: 5.01%, M: 7.43%) were significantly greater in the mixed plantation, whereas the relative abundances of Inocybe (P: 12.34%, M: 5.14%) and Clavulicium (P: 2.41%, M: 0.24%) were greater in the pure plantation (Figure 3c,d). The relative abundance of Solicoccozyma was also significantly different between the stand types (P: 5.03%, M: 2.17%).

**Figure 4:**
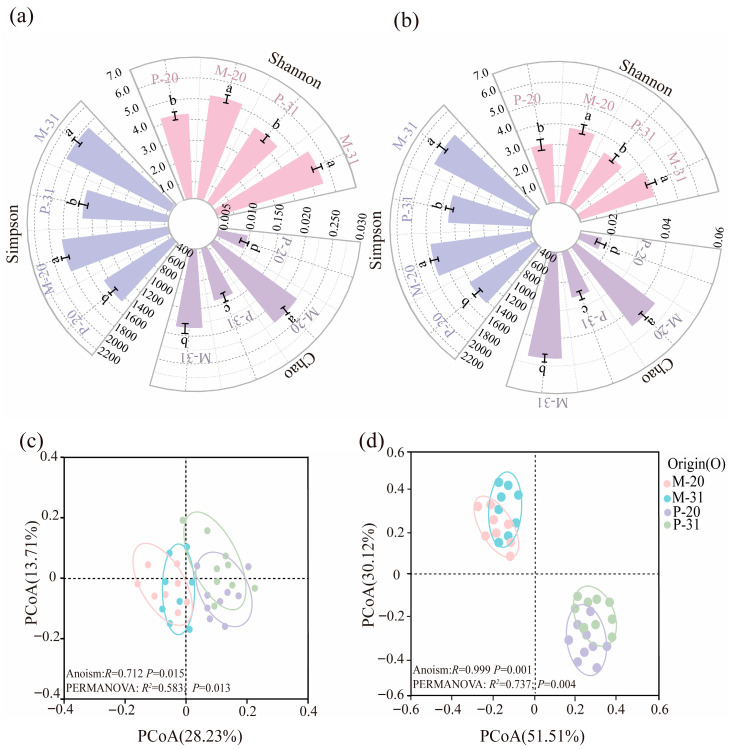
Bacterial and fungal diversity and PCoA analysis of *L. olgensis* rhizosphere. Note: (**a**,**b**) represents Polar of Simpson, Chao and Shannon index. (**a**) Bacteria; (**b**) fungi. Different letters above the boxes indicate a significant difference. (**c**,**d**) Principal Coordinate Analysis (PCoA) for bacterial and fungal based on P and M.  In (**c**,**d**), Dashed lines indicate the approximate separation boundaries between sample groups based on the PCoA ordination.

Diversity analysis revealed that the alpha diversity indices (*Shannon*, *Simpson*, and Chao1 indices) of both the bacterial and fungal communities in the *L. olgensis* rhizosphere were significantly greater in the mixed plantation than in the pure plantation (Figure 4a,b). Principal coordinate analysis (PCoA) based on Bray–Curtis distances indicated clear separations between the bacterial and fungal communities of the pure (P) and mixed (M) plantations (*p* < 0.05) (Figure 4c,d). The PCoA results revealed that compared with bacterial communities, fungal communities explained a greater proportion of the variation between pure and mixed plantations (*R*^2^ = 73.7%, *p* = 0.004) (*R*^2^ = 58.3%, *p* = 0.013).

### 3.3. Changes in the Rhizosphere Soil Microbial Cross-Kingdom Network and Assembly Process

**Figure 5:**
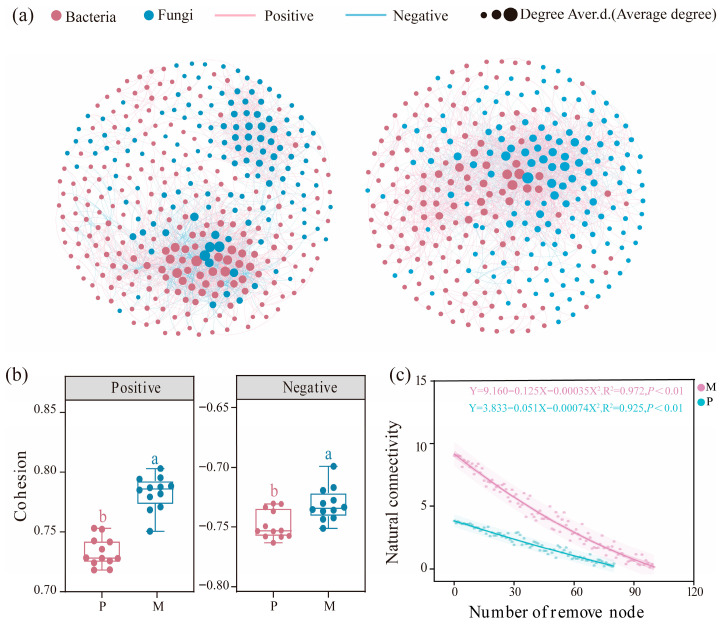
Bacterial–fungal co-occurrence networks based on all samples of *L. olgensis* rhizosphere soil in pure forest (left) and mixed forest (right). Note: (**a**) A connection stand represents a significant (*p* < 0.05) correlation between two OTUs. The size of each node is proportional to the number of connections. (**b**) Bacterial–fungal interdomain network cohesion was analyzed in both bulk soil and rhizosphere compartments. Positive cohesion values, derived from pairwise positive correlations, indicate the extent of microbial cooperation within the community. Different letters above the boxes indicate a significant difference. (**c**) Network stability was evaluated using natural connectivity metrics.

In the *L. olgensis* rhizosphere soil, positive correlations between bacteria and fungi predominated in the cross-kingdom network (Figure 5a). The proportions of positive associations for M and P were 73% and 61%, respectively. Similarly, the number of bacterial nodes increased markedly by 40.1%, substantially exceeding the 6.1% increase observed for fungal nodes. Tree species mixing significantly increased network topological properties, including the number of nodes, average path length, average degree, clustering coefficient, and modularity (*p* < 0.05). Specifically, the average degree increased by 69.4%, the modularity increased by 59.2%, and the clustering coefficient increased by 35.3%, whereas the average path length decreased by 36.7% (Figure S3). Furthermore, both positive and negative cohesiveness were significantly greater in the M network than in the P network (*p* < 0.01) (Figure 5b), indicating greater robustness of the M network. Natural connectivity analysis demonstrated that the M network maintained higher connectivity as nodes were progressively removed (Figure 5c). On the basis of within-module connectivity (Z_i_) and among-module connectivity (P_i_), 24 keystone taxa crucial to network structure were identified, including 7 module hubs and 6 connectors for bacteria and 4 module hubs and 7 connectors for fungi (Figure S4).

**Figure 6:**
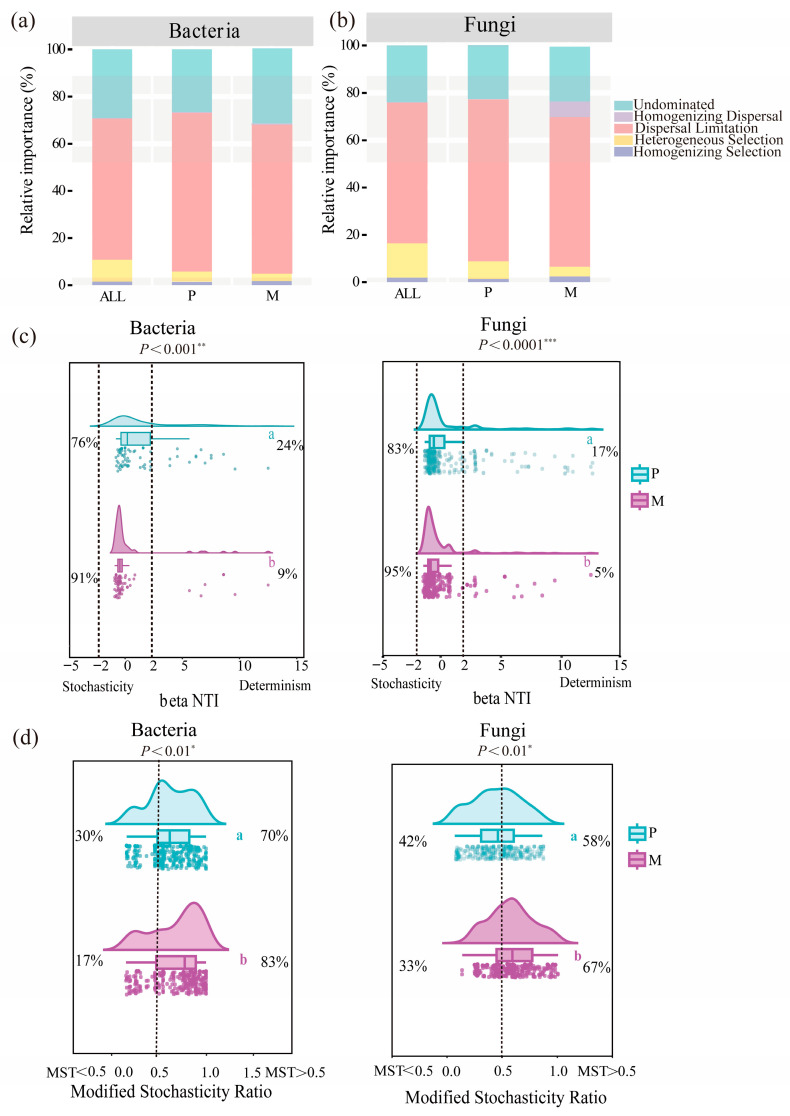
The ecological processes of *L. olgensis* rhizosphere soil bacterial and fungal communities. Note: (**a**,**b**) Relative importance of five ecological processes in pure (P) and mixed (M) plantations, based on the beta nearest taxon index (βNTI) and Bray-Curtis-based Raup-Crick index (RCBray). (**c**) Phylogenetic turnover (βNTI) and the proportion of deterministic (|βNTI| > 2) versus stochastic (|βNTI| < 2) processes. (**d**) Modified stochasticity ratio (MST) of bacterial communities. Significant differences (*p* < 0.05) between P and M are indicated by different letters. *, ** and *** exhibit significance at the levels of *p* < 0.01, *p* < 0.001 and *p* < 0.0001, respectively.

We utilized the beta nearest taxon index (*β*NTI) and the Bray–Crick-based Raup–Crick metric (RCBray) to infer the assembly processes of bacterial and fungal communities in the *L. olgensis* rhizosphere of pure and mixed plantations. The results indicated that stochastic processes primarily governed the assembly of *L. olgensis* rhizosphere microbial communities (|*β*NTI| < 2), with homogenizing dispersal and dispersal limitation being particularly influential (Figure 6a,b). With respect to bacteria, dispersal limitation in P (69.0%) exceeded that in M (54.7%), and a similar pattern was observed for fungi, with P (73.9%) being higher than M (55.6%) (Figure 6a,b). The proportion of community assembly controlled by stochastic processes was lower in P (83% for bacteria, 76% for fungi) than in M (95% for bacteria, 87% for fungi) (Figure 6c). Additionally, the modified stochasticity ratio (MST) analysis revealed that the contribution of stochastic processes was greater in M (bacteria: 83%, fungi: 67%) than in P (bacteria: 70%, fungi: 58%) (MST > 0.5) (Figure 6d), further indicating a greater influence of stochastic processes in the mixed plantation.

The Sloan neutral community model (NCM) revealed that the relative contribution of stochastic processes to bacterial community assembly was 50.7% in P and 71.9% in M (Figure S5a–d). With respect to the fungal communities, the stochastic contribution was 36.7% in P and 49.6% in M. The estimated migration rates (m) were higher in M (bacteria: 1.232; fungi: 0.3373) than in P (bacteria: 0.95334; fungi: 0.1945), suggesting stronger microbial dispersal potential in the mixed plantation. Furthermore, the higher goodness-of-fit (*R*^2^) of the NCM in M (bacteria: 0.719; fungi: 0.496) than in P (bacteria: 0.507; fungi: 0.367) indicated a greater influence of stochastic processes on microbial community assembly in the mixed plantation.

### 3.4. Influence of Rhizosphere Microbial Properties on SMC

**Figure 7:**
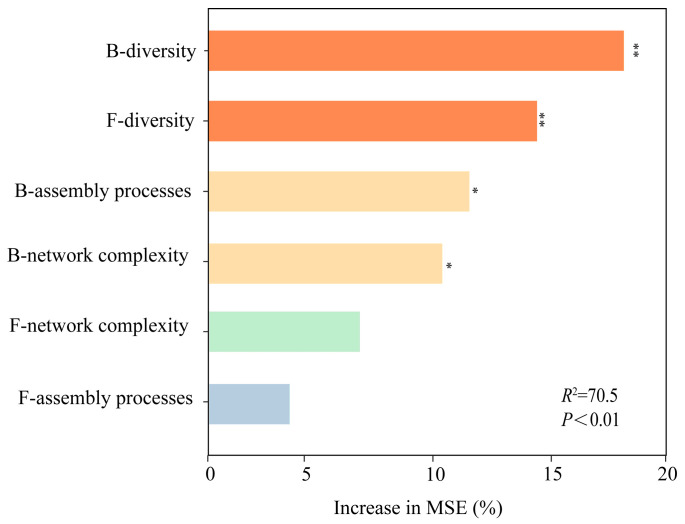
Potential microbial community drivers of SMC in rhizosphere soil of *L. olgensis.* Note: The relative significance of each predictor was assessed by calculating the percentage increase in mean squared error (%lncMSE), with greater values indicating higher variable importance. The microbial predictors were designated with prefixes: “F_” representing fungal taxa and “B_” denoting bacterial taxa. Quantitative analysis of microbial biomass was performed through real-time PCR quantification, with data normalized by log10-transforming the gene copy numbers per gram of dry soil. * and ** exhibit significance at the levels of *p* < 0.05 and *p* < 0.01, respectively.

**Figure 8:**
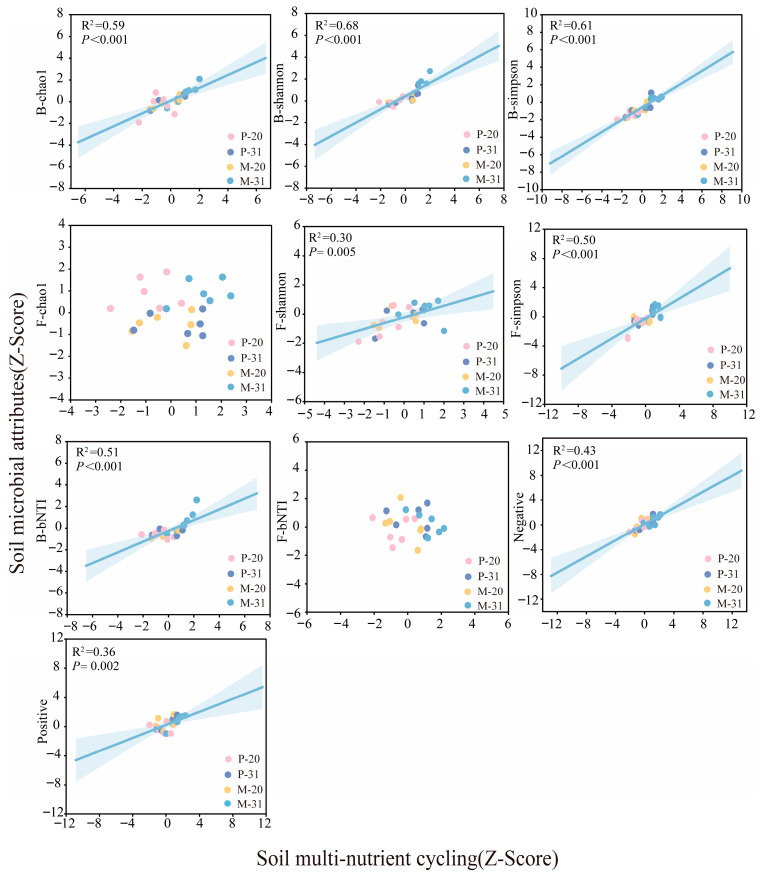
The ordinary least-squares regression showing the links between SMC and microbial communities.

Random forest analysis was used to assess the predictive power of microbial properties for SMC. The diversity indices of both the bacterial and fungal communities in the mixed plantation rhizosphere explained the most SMC variation, followed by network complexity and bacterial assembly processes (Figure 7). The relationships between the soil microbiome (i.e., the α diversity of bacteria and fungi, bacterial assembly and network complexity) and SMC (Figure 8) were further evaluated via correlation analyses and OLS. In the stand, SMC was significantly and positively associated with bacterial α-diversity (Chao1, *R*^2^ = 0.59; Shannon, *R*^2^ = 0.68; Simpson, *R*^2^ = 0.61; Figure 8) and fungal α-diversity (Shannon, *R*^2^ = 0.30; Simpson, *R*^2^ = 0.50; Figure 8) and was also significantly positively linked to bacterial assembly processes (*R*^2^ = 0.51; Figure 8). Similarly, SMC was significantly correlated with network complexity (negative associations: *R*^2^ = 0.43; positive associations: *R*^2^ = 0.36; Figure 8). Moreover, pH and SWC were both strongly and positively correlated with bacterial α-diversity (*p* < 0.001; Figures S6 and S7). The pH showed a highly significant positive relationship with fungal α-diversity, bacterial assembly processes, and positive associations (*p* < 0.001; Figure S6), but was significantly negatively correlated with negative associations (*p* < 0.001; Figure S6).

**Figure 9:**
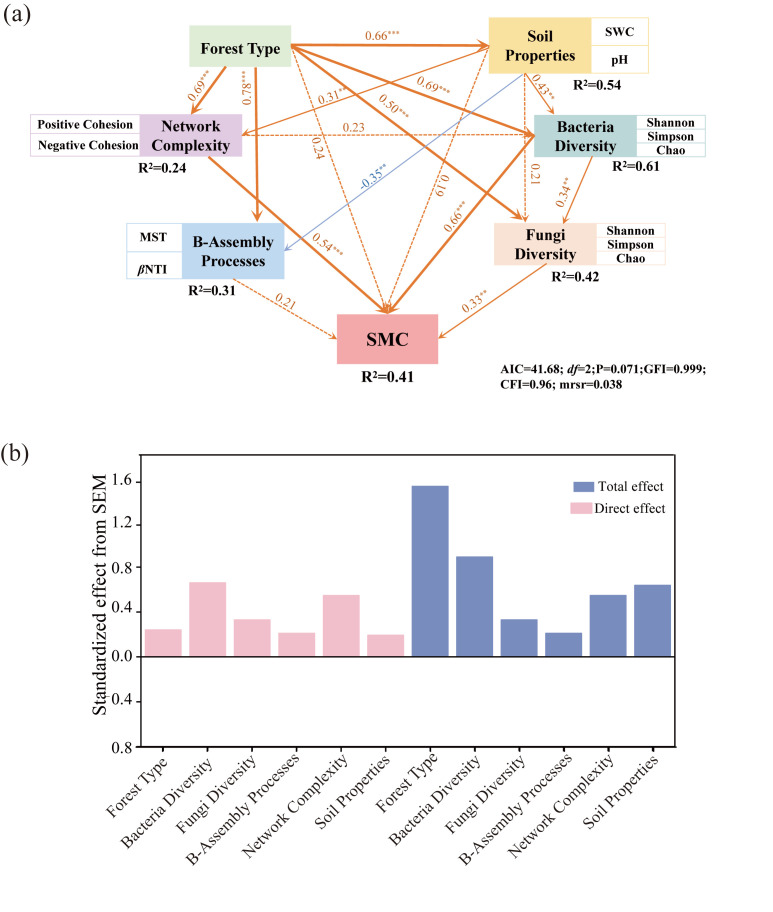
Main drivers of *L. olgensis* rhizosphere SMC and their action pathways. Note: Structural equation models (SEMs) accounting for the direct and indirect effects of forest type, soil properties, bacterial and fungal diversity, bacterial assembly processes and network complexity on SMC (**a**,**b**). The blue and orange arrows respectively represent positive and negative influences. The width of the arrow is related to the path coefficient. SWC, soil water content; SMC, soil multi-nutrient cycling, B-Assembly Processes, bacterial assembly processes, MSE, mean square error. *R*^2^ indicates the total variation in the dependent variable explained by all the independent variables. ** and ***  exhibit significance at the levels of *p* < 0.01 and *p* < 0.001, respectively.

We constructed structural equation models (SEMs) separately for fungi and bacteria to elucidate the regulatory relationships among these factors (Figure 9a,b). The final model explained 41% of the variation in SMC. The forest type had an indirect positive effect on SMC by directly enhancing the soil properties, bacterial assembly process, network complexity, bacterial diversity, and fungal diversity, with a standardized total effect (STE) of 1.52 according to the SEM (Figure 9a,b). Specifically, the direct positive effects on SMC were attributed to soil properties (STE = 0.19), bacterial diversity (STE = 0.66) and fungal diversity (STE = 0.33). Analysis of the relationships between network complexity, bacterial assembly processes, and SMC under the influence of stand type and soil properties (Figure 9a,b) indicated that bacterial assembly processes (STE = 0.21) and network complexity (STE = 0.54) positively affected SMC. Notably, microbial diversity positively influenced SMC indirectly by driving network complexity (Figure 9a,b). In summary, among the various drivers evaluated, microbial diversity emerged as the most significant contributor to SMC (Figure 9a,b).

## 4. Discussion

### 4.1. Microbial Diversity in the Rhizosphere Soil

Soil microbial communities are central to decomposition, nutrient cycling, and the maintenance of soil health, yet their contributions vary strongly across environmental contexts [39]. Tree species mixing significantly altered the composition of the rhizosphere microbial community in *L. olgensis* (Figure 3 and Figure 4) and substantially increased the microbial α diversity (*p* < 0.01; Figure 5), thereby supporting our first hypothesis. These findings are consistent with those of previous studies showing that changes in tree species composition can influence microbial community structure by altering key soil properties, such as pH and SWC [40,41]. This study further demonstrated that tree species mixing significantly affects microbial community composition by altering rhizosphere soil properties (notably soil moisture and pH) in the rhizosphere of *L. olgensis*, although its effects on bacterial and fungal diversity differ substantially (Figures S6 and S7). Specifically, increases in soil pH were associated with significant increases in both bacterial and fungal diversity, whereas higher SWC was associated only with increased bacterial diversity and had no significant effect on fungal diversity (Figures S6 and S7). Previous studies have reported that increasing soil pH from acidic (pH < 5.0) to near-neutral (pH 6.0–7.0) significantly increases the species richness of both bacteria and fungi [42]. In contrast, the effects of soil water content on bacterial and fungal diversity differ markedly. Bacterial α diversity is highly sensitive to SWC, whereas fungal diversity responds weaklier (Figure S7). This divergence may be due to the distinct physiological adaptation strategies of bacteria and fungi. Bacteria largely depend on thin water films in soil for nutrient diffusion, rendering their activity highly sensitive to SWC [43]. In contrast, through extensive hyphal networks, fungi can translocate water and nutrients across micro-environments [44]. This structural advantage may confer greater resilience of fungi to water content variation, such that fungal community diversity is more strongly influenced by other factors, such as soil pH [45]. Furthermore, bacterial diversity contributed the most to the increase in the SMC, followed by fungal diversity, as supported by the SEM results (Figure 8), which is consistent with the findings of previous studies [42]. This research highlights that a mixture of tree species promotes microbial diversity through SWC and pH in *L. olgensis* rhizosphere soil, which subsequently influences the SMC.

### 4.2. Rhizosphere Bacteria–Fungi Kingdom Network and Assembly Processes

The assembly of microbial communities is typically governed by the combined effects of deterministic processes (e.g., environmental filtering and species interactions) and stochastic processes (e.g., dispersal limitation and ecological drift) [46]. This can result in more diverse and dynamic microbial communities capable of adapting to varying environmental conditions [47]. Our study revealed that stochastic processes dominated the assembly of the *L. olgensis* rhizosphere microbial communities, with their influence being significantly stronger in the mixed plantation than in the pure plantation (Figure 6). This aligns with findings in Chinese fir plantations, suggesting that tree species mixing reduces environmental filtering and increases the influence of stochasticity [48]. The enhanced role of stochasticity following mixing may be linked to elevated rhizosphere nutrient levels (Figure 1). Improvements in rhizosphere soil properties in mixed stands (e.g., soil water content and pH) can increase soil nutrient availability (Figure S2), resulting in the supply of microbes with a more diverse resource base, substantially broadening the width of the habitat niche and thereby weakening environmental filtering [49]. Furthermore, microbial morphological traits can serve as important indicators of dispersal ability [50]. Consistent with the ‘size-plasticity’ and ‘size-dispersal’ hypotheses, fungi are more susceptible to dispersal limitation than bacteria are [51]. Similarly, we found that compared with bacterial communities, fungal communities in the *L. olgensis* rhizosphere were subjected to stronger dispersal limitations (Figure 6). Tree species mixing alleviated dispersal limitation for bacteria (Figure S5), potentially related to the theory of niche preemption, where populations with higher fitness are more likely to successfully colonize and exhibit increased dispersal ability [50]. From a functional perspective, stochastically assembled communities can buffer environmental disturbances through mechanisms such as metabolic redundancy and functional complementary [52], thereby enhancing ecosystem functioning. Notably, our study revealed a link between bacterial assembly processes and SMC (Figure 9), suggesting that the increased stochasticity of bacteria across habitats enhances their functional contribution to the community, highlighting the critical role of bacterial assembly mechanisms in maintaining terrestrial soil nutrient cycling.

Microbial co-occurrence networks play a vital role in maintaining community stability; however, these networks have received insufficient attention in studies on tree species mixing. A recent study on mixed eucalyptus plantations revealed that mixing significantly increased the complexity of cross-domain fungal and bacterial networks [53]. Our findings further support this conclusion, demonstrating that the microbial network in the *L. olgensis* rhizosphere of the mixed plantation contained more nodes and edges (Figure S3) and was therefore more complex and stable than that in the pure plantation. Concurrently, tree species mixing was associated with a higher proportion of positive correlations and reduced network vulnerability, indicating altered patterns of microbial co-occurrence within the bacteria–fungi cross-domain network (Figure 5). These patterns may be indirectly related to increased carbon inputs from *L. olgensis* roots under mixed plantations [54], which can modify rhizosphere nutrient availability and potentially promote more frequent microbial interactions [55]. Notably, we found that the cross-domain bacterial–fungal network in the mixed plantation was significantly correlated with SMC (Figure 9), a phenomenon also reported in other ecosystems [6,42]. On the one hand, higher network complexity is commonly interpreted as reflecting tighter microbial associations and greater structural organization, which may be linked to enhanced resource turnover and nutrient cycling efficiency at the community level [20,56]. On the other hand, complex networks are generally considered less vulnerable to external disturbances due to their higher degree of inter-connectivity [19,42]. Therefore, in-depth investigations of the dynamics of microbial interaction networks and their contributions to maintaining soil health remain central topics in research on plantation ecosystem restoration.

### 4.3. Key Drivers of Rhizosphere SMC

In this study, microbial diversity emerged as the primary predictor of SMC, with greater explanatory power than stand type, soil properties, or network complexity did (Figure 9). Specifically, microbial diversity maintained a significant positive relationship with SMC (Figure 9), which persisted when multiple predictors were considered simultaneously (Figure 7, Figure 8 and Figure 9), thereby confirming our second hypothesis. As a crucial regulator of SMC, soil microbial diversity is modulated by nutrient availability and microenvironmental conditions [57], whereas increased tree species richness promotes soil heterogeneity and consequently sustains soil biodiversity [1]. Furthermore, higher microbial diversity facilitates richer metabolic capabilities, strengthens microbial interactions (e.g., cross-feeding), and improves nutrient cycling efficiency [58], collectively optimizing soil nutrient availability. Notably, microbial diversity also indirectly promoted SMC by enhancing network complexity (Figure 7, Figure 8 and Figure 9), indicating that network complexity reinforces the microbial diversity-SMC relationship. Previous studies have established network complexity as a critical dimension for predicting ecosystem functioning [19,20,59]. Thus, increased microbial diversity fosters stronger ecological connections among microbial taxa, yielding more complex network architectures that subsequently increase SMC. However, when stand composition, soil properties, and microbial diversity were considered together, network complexity had no significant direct effect on SMC (Figure 9), suggesting that its influence is strongly mediated by stand characteristics, soil physicochemical properties, and microbial diversity. Additionally, deterministic processes may dampen the effects of microbial diversity on SMC through dilution effects [60], and the predominant stochastic assembly of bacterial communities in our study mitigated such dilution (Figure 6) and strengthened the relationships between the microbial community and SMC (Figure 9). Collectively, these findings highlight the importance of microbial network complexity and assembly processes in sustaining SMC, advancing our understanding of biodiversity–ecosystem functional relationships in multi-nutrient contexts, with implications for soil health assessment, biodiversity conservation, and maintenance of multi-functionality in managed forest ecosystems. Consequently, future studies should focus on plant–soil microbe interactions across trophic levels to elucidate the integrated microbial mechanisms driving SMC, thereby facilitating comprehensive soil nutrient management in forest ecosystems.

In summary, our findings demonstrate that tree species mixing enhances soil multi-nutrient cycling (SMC) primarily by promoting more complex microbial networks and increasing the stochasticity of community assembly. These results elucidate key microbial mechanisms through which mixed-species plantations sustain multiple ecological functions and provide a mechanistic basis for managing soil health and biodiversity in forest ecosystems. Nevertheless, the temporal and spatial scope of this study imposes certain limitations. Future research should therefore incorporate long-term and multi-season monitoring to capture temporal dynamics, expand across broader spatial gradients to test the generality and context dependency of tree species mixing effects, and account for land-use history and legacy effects. Moreover, integrating observational studies with controlled experiments across diverse conifer–broadleaf species combinations, together with functional approaches such as metagenomics, metatranscriptomics, isotopic tracing, and enzyme activity profiling, will further strengthen causal inference and elucidate the mechanistic links between microbial diversity, functional potential, and soil multi-nutrient cycling.

## 5. Conclusions

This study demonstrated that mixed planting with broadleaf species in *L. olgensis* stands enhances SMC through modifications to soil properties, microbiome characteristics (diversity, network complexity, and assembly processes) and their interrelationships. Compared with pure stands, mixed stands promoted r-strategist microbial communities, increased diversity, enhanced stochastic assembly, and developed more complex microbial networks. Our analyses revealed consistent positive associations between SMC and improvements in soil properties, bacterial and fungal diversity, stochastic processes, and microbial network complexity. Specifically, microbial diversity, assembly processes, and soil properties were identified as direct drivers of SMC in *L. olgensis* forests. Importantly, tree species mixing enhanced SMC by modulating soil–microbial community interactions and indirectly through microbial diversity-mediated increases in network complexity. These findings collectively emphasize the ecological advantages of mixed conifer–broadleaf forests from microbial diversity, assembly, and network perspectives while underscoring their significance for enhancing soil microbial functionality. Furthermore, this study contributes to our understanding of how multispecies afforestation enhances forest ecosystem services through improved soil nutrient cycling.

## Data Availability

The data presented in this study are available on request from the corresponding author. The data are not publicly available due to containing unpublished research findings that are part of an ongoing study.

## Supplementary Materials

The following supporting information can be downloaded at: https://www.mdpi.com/article/10.3390/microorganisms14020388/s1, Table S1: Stand characteristics of pure and mixed plantations in this study; Table S2: Rhizosphere soil chemical properties of *L. olgensis* in pure and mixed plantations; Figure S1: Rhizosphere soil properties of *L. olgensis* in pure and mixed stands in temperate China; Figure S2: Relationships between soil water content (a) and pH (b) and SMC; Figure S3: Parameters in bacterial–fungal co-occurrence; Figure S4: Zi-Pi diagrams of bacteria and fungi based on the topological role of OTUs; Figure S5: The fit of the neutral community model (NCM) of bacterial (a,b) and Fungal (c,d) community assembly; Figure S6: Links between soil pH and soil microbial attributes; Figure S7: Links between soil water content and soil microbial attributes. References [61,62,63,64,65,66,67,68,69,70,71,72,73] are cited in the supplementary materials.
