# Supplementary material for: Tree Species Mixing Regulates Soil Multi-Nutrient Cycling by Altering Microbial Network Complexity and Assembly Processes in Larix olgensis
Source: Microorganisms. 2026 Feb 6;14(2):388. doi: 10.3390/microorganisms14020388 (PMC12943710; doi:10.3390/microorganisms14020388)
Supplement: Supplementary file 1 [file microorganisms-14-00388-s001.zip › microorganisms-4126325-supplementary.pdf]

Article

Tree Species Mixing Regulates Soil Multi-Nutrient Cycling by Altering Microbial Network Complexity and Assembly Processes in *Larix olgensis*

Yue Liu [1], Chunjing Jiao [1], Wanju Feng [2], Yuchun Yang [1,*], Bing Yang [2,*], Fang Wang [1] and Jun Wang [1]

1 Jilin Provincial Academy of Forestry Sciences, Changchun 130033, China; yueliu0211@163.com (Y.L.)
2 The Conservation of Endangered Wildlife Key Laboratory of Sichuan Province, Sichuan Academy of Giant Panda, Chengdu 610081, China
* Correspondence: yang-yu-chun@163.com (Y.Y.); xinyuan890@163.com (B.Y.)

1 Soil physicochemical analyses

The soil organic carbon (SOC) content was quantified via the Walkley-Black method[61]. Total phosphorus (TP) was quantified colorimetrically via a UV spectrophotometer (TU-1950, Puxi Ltd., Beijing, China) after wet digestion with $HClO_4$-H_2SO_4 and scandium colorimetry. Total nitrogen (TN) was measured using an elemental analyzer (Vario Max, Elementar, Germany). Available phosphorus (AP) was determined via the Olsen method[62]. The soil pH was measured via a pH meter (FE28, Shanghai) in a 1:2.5 soil-to-water suspension. The contents of soil nitrate-N (NO_3^--N) and ammonium-N (NH_4^+-N) were determined after the fresh soil was extracted with 2 M KCl, and the results were analyzed on a continuous-flow ion autoanalyzer (Scalar SANplus segmented flow analyser, Germany). The content of available potassium (AK) was determined via the EDTA titrimetric method via an inductively coupled plasma spectrometer (ICP-AES, Thermo, USA). The soil mass water content (SWC) was calculated when the soil was dried to a constant weight at 65°C.

2 Microbial Community Assembly Analysis

The Beta Nearest Taxon Index (βNTI) was calculated using the Picante package in R to infer community assembly processes for bacterial and fungal communities[63]. A null model was generated through 999 randomizations based on the observed data (OTU table and phylogenetic tree), and βNTI was computed as the deviation (in units of standard deviation) between the observed βMNTD and the βMNTD expected under the null distribution. Values of βNTI>2 indicate that deterministic processes predominate in shaping microbial community turnover, with βNTI<−2 and βNTI>2 representing homogeneous and heterogeneous selection, respectively[63]. When |βNTI| < 2, stochastic processes are considered the primary drivers of community variation. To further evaluate the relative influence of dispersal versus niche-based processes in community assembly, we calculated the dispersal-niche continuum index (DNCI) using the DNCI package[64]. The normalized stochasticity ratio (MST) was then computed using the NST package to quantify the relative contributions of deterministic (MST<0.5) and stochastic (MST>0.5) processes[65].

3 Bacterial-Fungal Cross-kingdom co-occurrence networks Analysis

Cross-kingdom co-occurrence networks for bacteria and fungi in the P and M stands were constructed using the IDENAP ecological network analysis workflow[66] in iNAP (https://onlinelibrary.wiley.com/doi/full/10.1002/imt2.13). Following the SparCC algorithm, associations with correlation coefficients r > 0.6 were retained to build cross-domain networks. Network nodes were categorized based on within-module

connectivity (Z_i) and among-module connectivity (P_i) into module hubs, peripherals, connectors, and network hubs following established criteria[67]. In accordance with prior studies[68], we quantified intra-domain associations (bacteria–bacteria and fungi-fungi edges) .The igraph package was used to calculate network complexity metrics, including the total number of nodes and links, and the average degree[69]. Network robustness was assessed using natural connectivity[70]. Furthermore, network complexity and vulnerability were evaluated under two node-removal scenarios: random removal of 50% of nodes and targeted removal of module hubs, following established methods[71]. Cohesion indices were calculated using a null model approach[72].

4 Random Forest Analysis and SEM Modeling

Spearman correlations between rhizosphere microbial communities and soil physicochemical properties were assessed using Mantel tests implemented in the linkET package. Random forest regression was conducted with the rfPermute and A3 packages to determine the significance of predictor variables and the overall model. Key bacterial and fungal genera contributing to shifts in conifer rhizosphere soils following mixed broadleaf introduction were identified using the Random Forest algorithm. Finally, The SEM was constructed using the piecewised SEM package to elucidate the relationships between root-associated microbiomes and soil properties[73].

Supplementary material captions

Table S1 Stand characteristics of pure and mixed plantations in this study.

Table S2 Rhizosphere soil chemical properties of *L.olgensis* in pure and mixed plantations.

Supplementary figures captions

Figure S1 Rhizosphere soil properties of *L.olgensis* in pure and mixed stands in temperate China.

Figure S2 Relationships between soil water content (A) and pH (B) and SMC.

Figure S3 Parameters in bacterial-fungal co-occurrence.

Figure S4 Zi-Pi diagrams of bacteria and fungi based on the topological role of OTUs.

Figure S5 The fit of the neutral community model (NCM) of bacterial(a,b) and Fungal(c,d) community assembly.

Figure S6 Links between soil pH and soil microbial attributes.

Figure S7 Links between soil water content and soil microbial attributes.

Table S1 Stand characteristics of pure and mixed plantations in this study.

Stand ages (years)	Forest Types	Diameter at breast height (cm)	Mean height (m)	Stem density (trees ha^{-2})	Canopy density (%)	Under-forest vegetation coverage rate (%)
21	P	11.62±1.03d	12.40±2.52	1564±61a	76.16±7.2	46.21±9.3
	M	13.60±2.42a	15.24±2.42	1483±58b	79.63±5.6	59.64±8.6
30	P	16.21±0.93d	18.62±2.78	1454±64a	68.41±4.5	54.64±8.2
	M	17.54±2.64ab	20.54±2.46	1364±67c	79.64±6.5	64.21±6.2

Note: P, the *L.olgensis* in pure plantations; M, the *L.olgensis* in mixed plantations; 20: 20-year-old stands; 31: 31-year-old stands. Different lowercase letters indicate significant differences among stands ($P < 0.05$).

Table S2 Rhizosphere soil chemical properties of *L.olgensis* in pure and mixed plantations

Soil Property	All		PF-21		PF-30	
	F	*P*	*F*	*P*	*F*	*P*
pH	28.2	**0.042***	16.7	**0.044***	21.7	**0.048***
SOC (g·kg^{-1})	46.8	**0.00****	49.4	**0.00****	39.7	**0.00****
TN (g·kg^{-1})	11.4	**0.00****	0.29	0.595	1.11	0.299
TP (g·kg^{-1})	2.7	0.32	2.18	0.402	6.5	0.256
NO_3^--N (mg·kg^{-1})	47.1	**0.00****	52.7	**0.00****	46.1	**0.00****
NH_4^+-N (mg·kg^{-1})	14.4	**0.041***	24.3	**0.037***	12.5	**0.042***
AK (mg·kg^{-1})	37.3	**0.00****	31.9	**0.00****	28.6	**0.015***
AP (mg·kg^{-1})	17.5	**0.042***	9.75	0.052	27.1	**0.029***
SWC (%)	5.17	0.28	5.12	0.461	7.15	0.148

Note: SOC, soil organic carbon; TN, Total nitrogen; TP, total phosphorus; NO_3^--N, nitrate nitrogen; NH_4^+-N, ammonium nitrogen; AK, available potassium, Available phosphorus; SWC, soil mass water content; *, P<0.05; **, P<0.01. Same below.

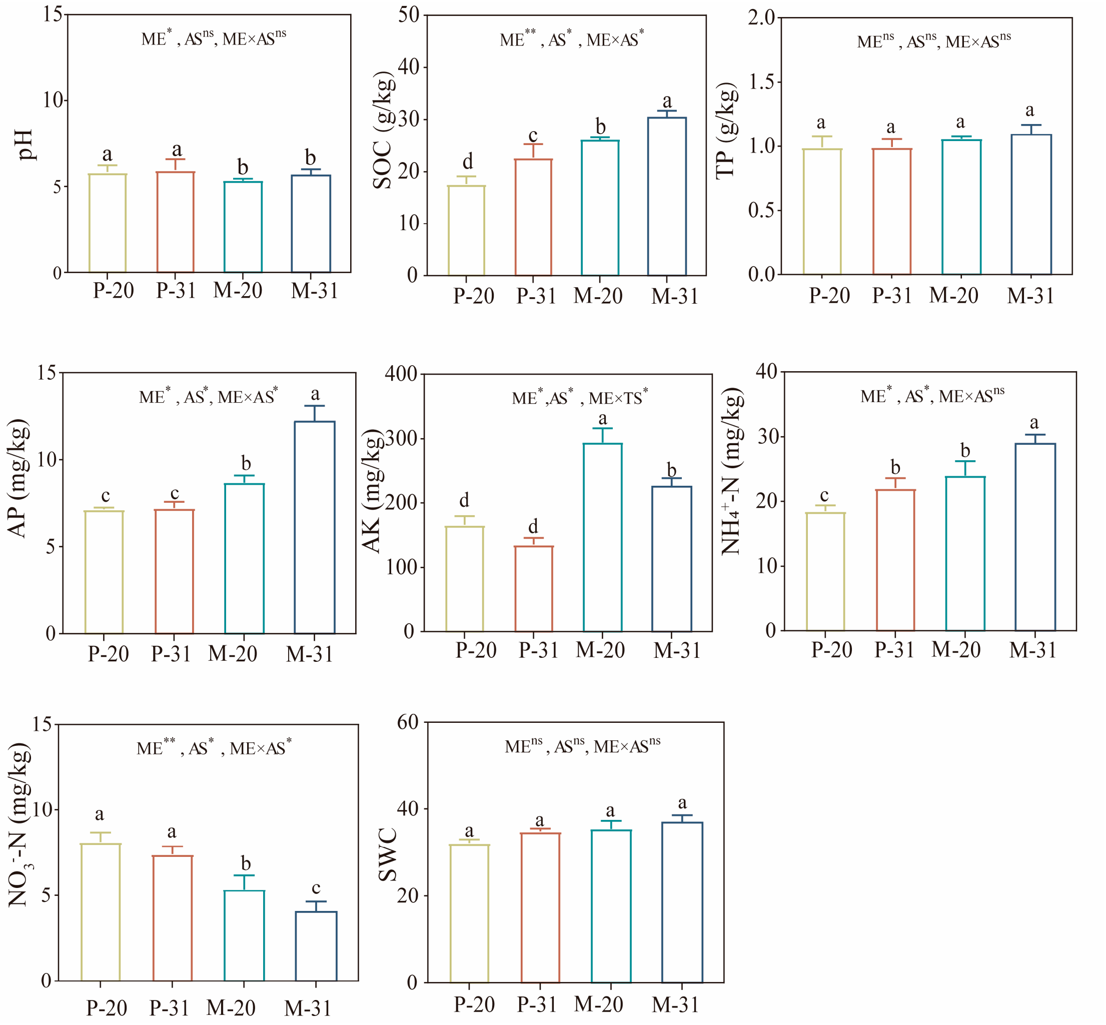

Figure S1 Rhizosphere soil properties of *L.olgensis* in pure and mixed stands in temperate China. Note: P, the *L.olgensis* in pure plantations; M, the *L.olgensis* in mixed plantations; 20: 20-year-old stands; 31: 31-year-old stands. Different lowercase letters indicate significant differences among the four forest types. The two-way ANOVA results are presented as an insert. ME: mixed effect. AE: stand age effect. * $p < 0.05$; ** $p < 0.01$; *** $p < 0.001$; NS not significant. Same below.

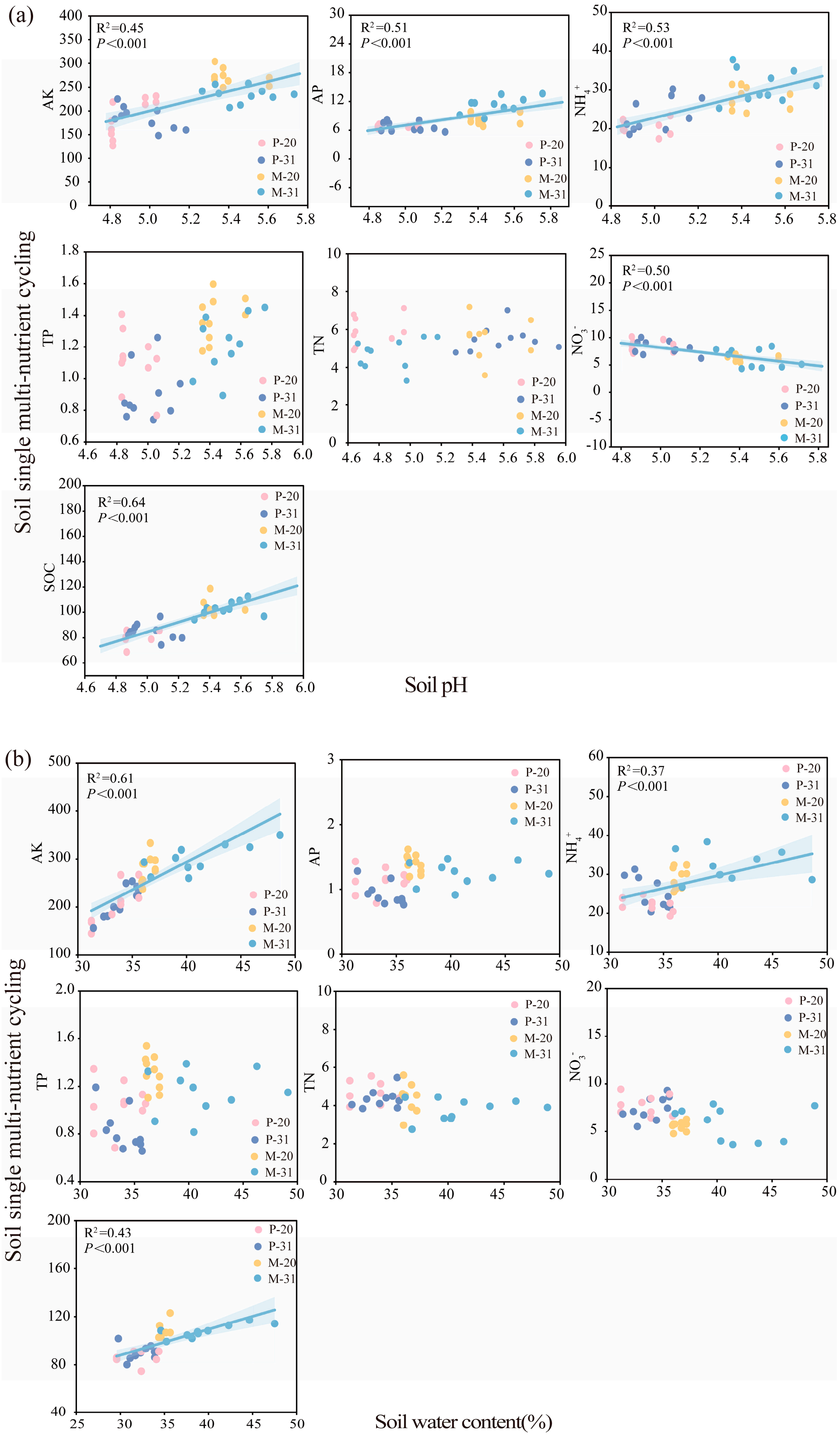
(a)
Soil single multi-nutrient cycling
AK
R²=0.45
P<0.001
AP
R²=0.51
P<0.001
NH4+
R²=0.53
P<0.001
TP
TN
NO3-
R²=0.50
P<0.001
SOC
R²=0.64
P<0.001
P-20
P-31
M-20
M-31
Soil pH
(b)
Soil single multi-nutrient cycling
AK
R²=0.61
P<0.001
AP
NH4+
R²=0.37
P<0.001
TP
TN
NO3-
R²=0.43
P<0.001
P-20
P-31
M-20
M-31
Soil water content(%)

Figure S2 Relationships between soil water content (a) and pH (b) and SMC.

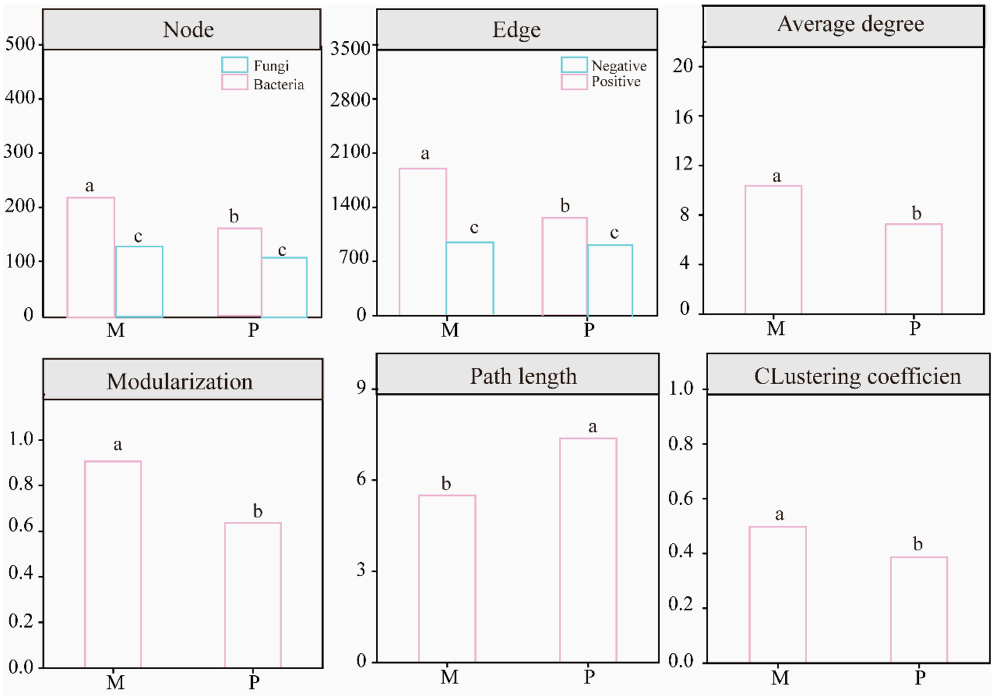

Figure S3. Parameters in bacterial-fungal co-occurrence. Note: Different lowercase letters and uppercase letters mean notable distinctions (*P*<0.05) of individual topological properties among different treatments in bulk soil and rhizosphere soil, respectively.

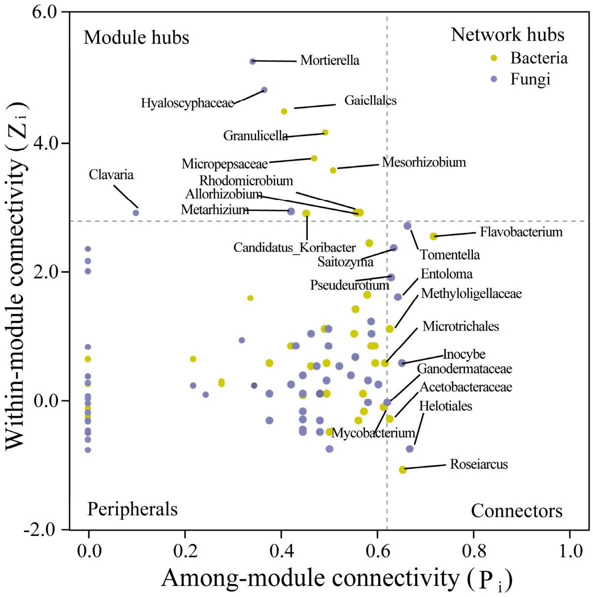

Figure S4 Z_i-P_i diagrams of bacteria and fungi based on the topological role of OTUs. Note: the bacterial and fungal networks have thresholds of 2.5 and 0.62 for OTUs classification, respectively.

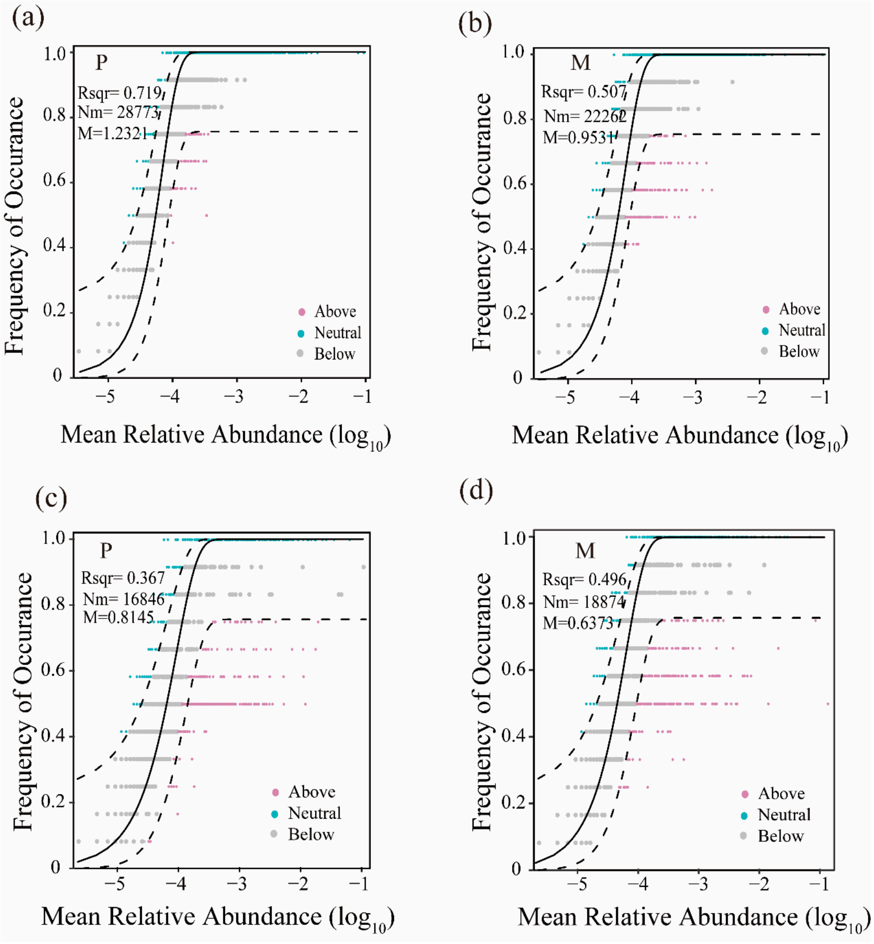

Figure S5 The fit of the neutral community model (NCM) of bacterial(a,b) and Fungal(c,d) community assembly.

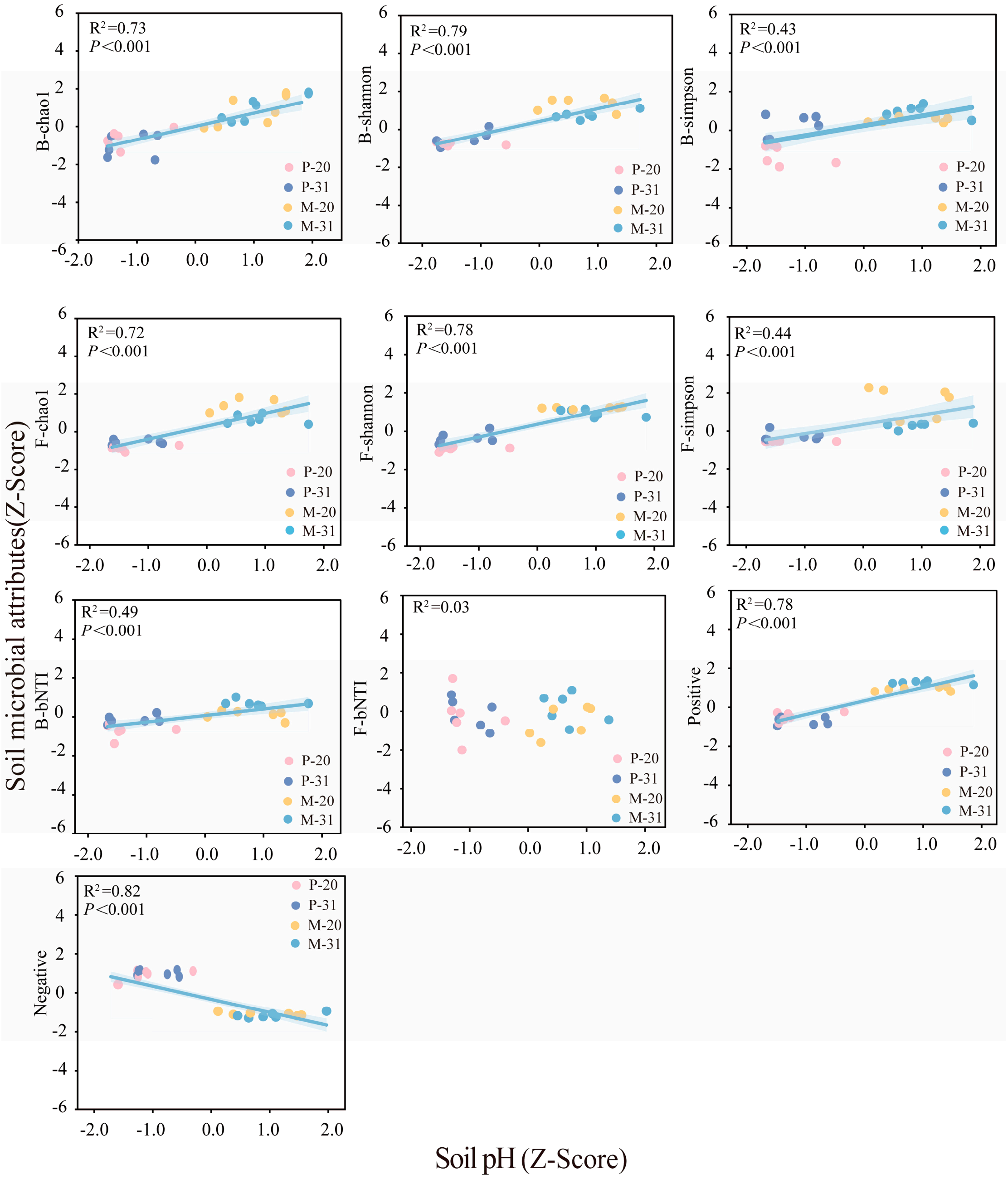

Figure S6 Links between soil pH and soil microbial attributes.

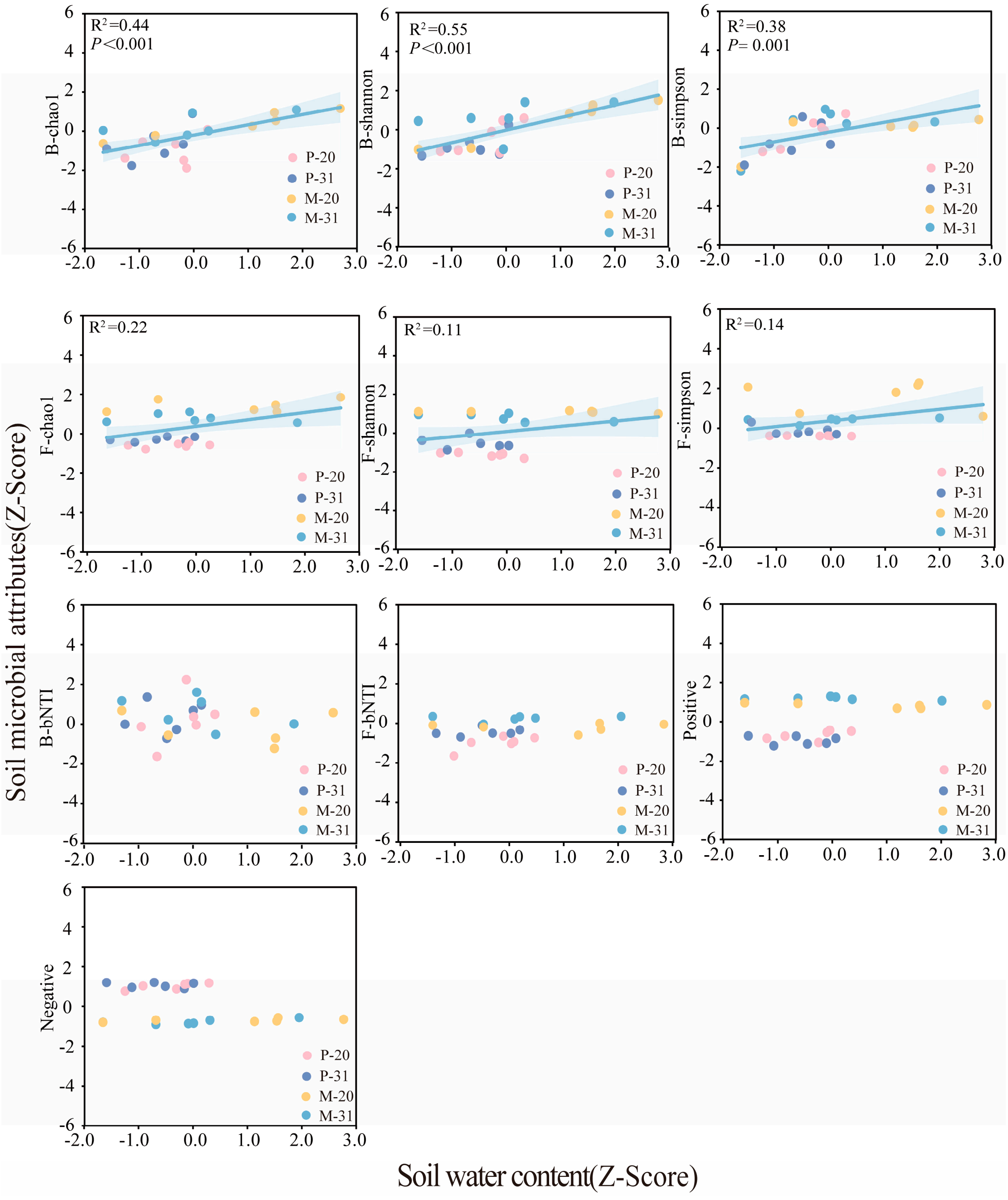

Figure S7 Links between soil water content and soil microbial attributes.
